# Aerobic prokaryotes do not have higher GC contents than anaerobic prokaryotes, but obligate aerobic prokaryotes have

**DOI:** 10.1186/s12862-019-1365-8

**Published:** 2019-01-28

**Authors:** Sidra Aslam, Xin-Ran Lan, Bo-Wen Zhang, Zheng-Lin Chen, Li Wang, Deng-Ke Niu

**Affiliations:** MOE Key Laboratory for Biodiversity Science and Ecological Engineering and Beijing Key Laboratory of Gene Resource and Molecular Development, College of Life Sciences, Beijing Normal University, Beijing, 100875 China

**Keywords:** Oxygen requirement, Reactive oxygen species, Aerobe, Anaerobe, Phylogenetically independent, Nucleotide composition, Guanine oxidation, Phylogenetic generalized least squares (PGLS) regression

## Abstract

**Background:**

Among the four bases, guanine is the most susceptible to damage from oxidative stress. Replication of DNA containing damaged guanines results in G to T mutations. Therefore, the mutations resulting from oxidative DNA damage are generally expected to predominantly consist of G to T (and C to A when the damaged guanine is not in the reference strand) and result in decreased GC content. However, the opposite pattern was reported 16 years ago in a study of prokaryotic genomes. Although that result has been widely cited and confirmed by nine later studies with similar methods, the omission of the effect of shared ancestry requires a re-examination of the reliability of the results.

**Results:**

When aerobic and obligate aerobic prokaryotes were mixed together and anaerobic and obligate anaerobic prokaryotes were mixed together, phylogenetic controlled analyses did not detect significant difference in GC content between aerobic and anaerobic prokaryotes. This result is consistent with two generally neglected studied that had accounted for the phylogenetic relationship. However, when obligate aerobic prokaryotes were compared with aerobic prokaryotes, anaerobic prokaryotes, and obligate anaerobic prokaryotes separately using phylogenetic regression analysis, a significant positive association was observed between aerobiosis and GC content, no matter it was calculated from whole genome sequences or the 4-fold degenerate sites of protein-coding genes. Obligate aerobes have significantly higher GC content than aerobes, anaerobes, and obligate anaerobes.

**Conclusions:**

The positive association between aerobiosis and GC content could be attributed to a mutational force resulting from incorporation of damaged deoxyguanosine during DNA replication rather than oxidation of the guanine nucleotides within DNA sequences. Our results indicate a grade in the aerobiosis-associated mutational force, strong in obligate aerobes, moderate in aerobes, weak in anaerobes and obligate anaerobes.

**Electronic supplementary material:**

The online version of this article (10.1186/s12862-019-1365-8) contains supplementary material, which is available to authorized users.

## Background

Oxygen is an essential environmental factor for most organisms living on Earth, and its accumulation was the most significant change in the evolution of the biosphere and dramatically influenced the evolutionary trajectory of all exposed organisms [1]. Oxidative metabolism provides a large amount of energy to aerobic organisms and produces an unavoidable by-product: reactive oxygen species (ROS). ROS are highly reactive with most cellular organic molecules, including nucleotides and their polymerized products, DNA and RNA. Among the four bases, guanine has the lowest oxidation potential and is the most susceptible to oxidation [2]. The direct products of deoxyguanosine oxidation are 8-oxo-7,8-dihydro-guanosine (8-oxoG) and 2,6-diamino-4-hydroxy-5-formamidopyrimidine. As 8-oxoG has a lower oxidation potential than deoxyguanosine, 8-oxoG is susceptible to further oxidation into several hyper-oxidized products [3]. The replication of DNA containing these damaged deoxyguanosines can cause G to T mutations, the frequency of which depends on the efficiency of DNA repair enzymes and the accuracy of replication enzymes [3]. When the oxidatively damaged guanines are not in the reference strand, the mutations they caused would manifest as C to A mutations in the reference strand. Therefore, in some literatures the mutations resulting from oxidatively damaged guanines were denoted by G to T transversions while in other literatures they were denoted by G:C to T:A transversions. No matter which means of presentation, the G:C to T:A transversions were generally considered the hallmark of oxidative damage to DNA [4–7]. Consequently, oxidative DNA damage was generally believed to be a mutational force to decrease GC content [8–10]. Consistent with this idea, a negative association had been observed between metabolic rate and the GC content at the silent sites of animal mitochondrial genomes [11].

However, 16 years ago, Naya et al. [10] observed an entirely opposite pattern in which aerobic prokaryotes had higher GC contents than anaerobic prokaryotes in a comparison of whole-genome GC content using nonphylogenetically controlled statistics. Furthermore, these authors showed that the pattern was still evident when aerobes and anaerobes were compared within each major phylum of archaea and bacteria. Opposing to the widespread belief that oxidative stress causes frequent G:C to T:A transversions and decreases GC content, this result was described as “*counterintuitive*” [8]. Naya et al. abandoned the neutralist interpretation to investigate possible selective forces, and they found that aerobes have lower frequencies of amino acids that are more susceptible to oxidation. As the non-synonymous sites of these amino acids are AT-rich, the high GC content of the aerobes might be explained by a deficiency of these amino acids. Moreover, they identified two potential benefits for aerobes with higher GC content. First, a high GC content might provide more stability to the DNA double strand, which would then be less accessible to oxygen radicals. Second, guanines located at synonymous sites might play a sacrificial role to protect other bases. This intriguing idea has been presented repeatedly [12, 13]. However, sacrificial guanine bases are easily mutated to T, and a mechanism is not available to maintain the sacrificial guanine bases during evolution [9]. In addition, the higher GC content of aerobes might be explained by another mutational force that have been generally overlooked. Guanine oxidation can occur not only within DNA strand but also before incorporation of the guanine nucleotide into DNA [14–16]. An oxidized guanine nucleotide is generally incorporated at the position of thymidine rather than guanine, which would cause T to G mutations in the next round of replication if the 8-oxoG happens to switch into the *anti* conformation. Seven years later, the same group found that the GC content of microbial communities living in the dissolved oxygen minimum layer (770 m) is lower than that of communities living in other (either below or above) layers of the seawater column in the North Pacific Subtropical Gyre, thus emphasizing the link between aerobiosis and genomic GC content [17]. In contrast, three later studies on seawater columns ranging from tens to thousands of metres observed that the GC content of metagenomes tends to increase linearly with depth in marine habitats, with the lowest GC content observed in near-surface stratified waters [18–20]. Regardless of the data obtained for microbial communities inhabiting different seawater depths, the pattern of higher GC content in aerobes has been repeatedly observed in various nonphylogenetically controlled comparisons. Later studies by nine independent groups, each with their own criteria for selecting species, observed the same pattern [21–29].

A possible explanation of the counterintuitive observations is provided by artefacts resulting from the phylogenetic non-independence of the data [30]. In 2008 and 2010, two groups independently compared the whole-genome GC content of aerobes and anaerobes and accounted for the phylogenetic relationships [24, 31]; however, they did not find a significant association between aerobiosis and GC content in the prokaryotic species they studied. These findings have received very little attention, which was likely because the two publications did not focus on the insignificant relationship between aerobiosis and GC content. Since 2009, the study by Naya et al. [10] has been cited 86 times (Google Scholar; access date: May 15, 2018); however, only one of the cited studies explicitly noted the conflicting results: “*oxygen requirement* [10] *may (or may not * [24]* ) have an impact on GC content*” [32]. The present study calls attention to these contradictory results. We took advantage of the rapid accumulation of sequenced genomes and performed an extensive investigation on the GC contents of aerobic and anaerobic prokaryotes using two phylogenetically controlled methods: 1) pairwise comparison of aerobes and their close anaerobic relatives and 2) phylogenetic generalized least squares (PGLS) regression.

## Results

### Nonphylogenetically controlled comparison showed higher GC content in aerobes

We first compared the genomic GC contents of the 1057 aerobic samples (including obligate aerobes) and the 1029 anaerobic samples (including obligate anaerobes) without considering their positions in the phylogenetic tree. The genomic GC contents of the aerobic samples and the anaerobic samples are 56.3% ± 12.6% and 45.9% ± 11.0%, respectively. Two-tailed Mann-Whitney *U* test showed that the difference between them is highly significant (*P* = 1.1 × 10^− 75^). Limiting this comparison within bacteria or archaea gave similar results (*P* = 7.6 × 10^− 61^ and 4.1 × 10^− 21^, respectively). Despite the much larger dataset, we also observed significantly higher GC content in aerobes than anaerobes. The reproducibility of this result is so high that the same pattern had been consistently observed in ten independent studies with nonphylogenetically controlled methods [10, 21–29].

### Pairwise comparison did not reveal significant difference

**Fig. 1:**
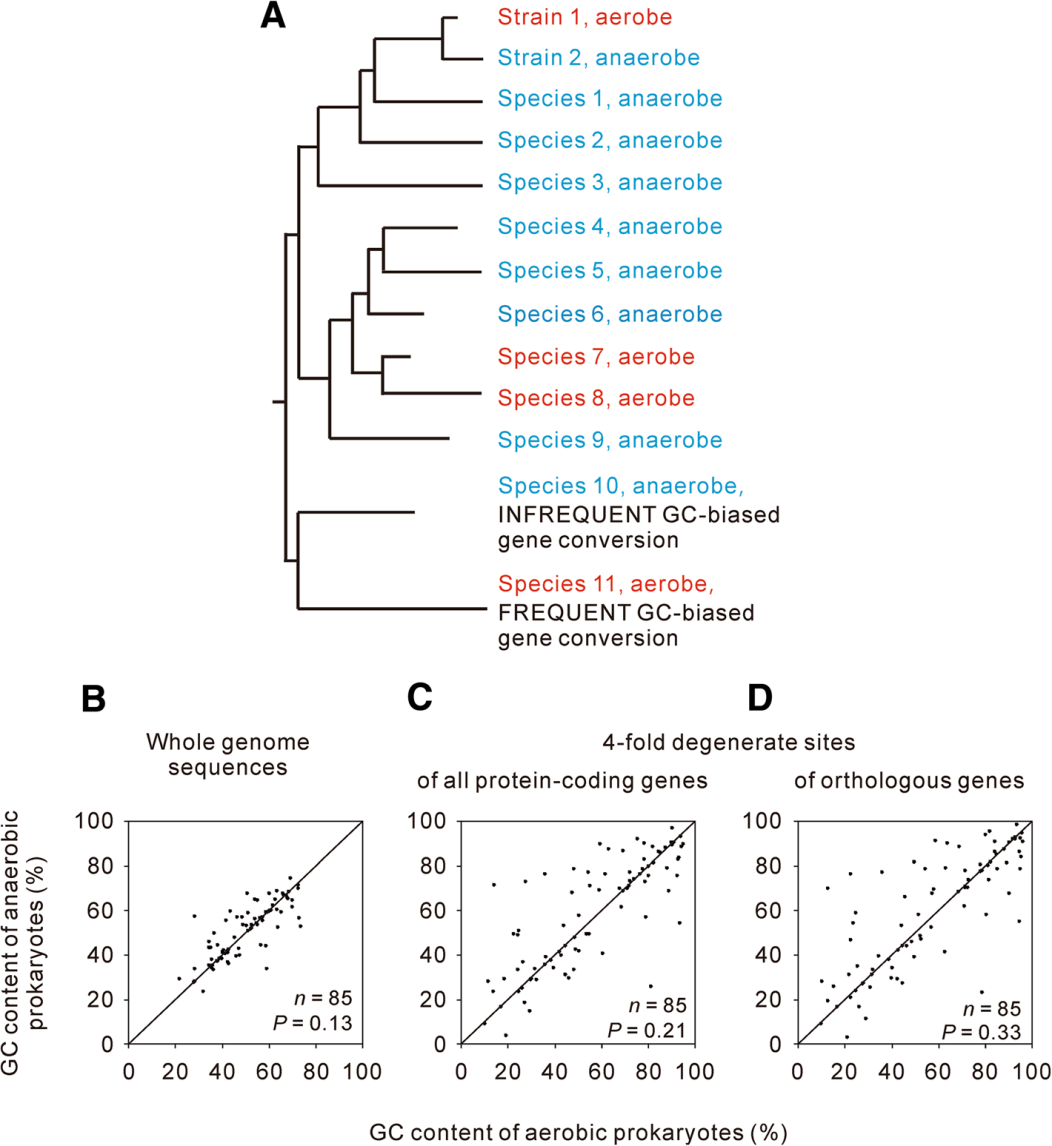
Pairwise comparison of GC content between aerobic and anaerobic prokaryotes. **a** Illustration of the difference between nonphylogenetically-controlled comparisons and phylogenetically-controlled comparison performed in this study. In a nonphylogenetically controlled comparison, the aerobes (including strain 1, species 7, species 8, and species 11) are compared to all the anaerobes (including strain 2, species 1–6, and species 9–10). However, only three changes in oxygen requirement are observed in the illustrated evolutionary tree. The differences in GC content between these three branches are likely to be associated with changes in the oxygen requirement. Therefore, only three pairs should be included in a phylogenetically controlled comparison. For branches having multiple strains/species with different evolutionary rates (e.g.*,* species 4–8), we paired the slowly evolved aerobic strain/species with the slowly evolved anaerobic strain/species (species 6 vs species 7). In cases with two or more strains/species with identical divergence times, we preferentially selected the genomes in which more genes had been annotated. Next, the comparisons were duplicated using the dataset including the quickly evolved pairs (e.g.*,* species 5 vs species 8 selected from species 4–8). Nearly identical results were obtained in the duplicated comparison. The results of the former are presented in Fig. 1b, c, and d and Table S1, and those of the latter are deposited as electronic supplementary material (Additional file 1: Figure S1 and Table S2). The choice of an anaerobe from species 4, 5 or 6 or an aerobe from species 7 or 8 did not alter the results. **b** Comparison of the GC content calculated from whole-genome sequences. **c** Comparison of GC content at the 4FDS of all protein-coding genes in each genome. **d** Comparison of GC content at the 4FDS of orthologous genes. The diagonal line represents cases in which aerobes and their paired anaerobes have the same GC content. Points above the line represent cases in which anaerobes have higher GC content than their paired aerobes, while points below the line indicate the reverse. All significance values were calculated using two-tailed Wilcoxon signed-rank tests

To control the effects of a common ancestor, we performed a pairwise comparison between aerobes and anaerobes that are adjacent in the phylogenetic tree (Fig. 1a). The difference in GC content within one pair is phylogenetically independent of the differences within any other pairs. Because of the limited number of pairs we obtained, obligate aerobes or anaerobes were not analysed separately. The aerobes and obligate aerobes were merged into one group termed aerobes, and the anaerobes and obligate anaerobes were merged into another group termed anaerobes. Pairwise comparisons of the GC content between the selected 85 aerobe-anaerobe pairs can thus be considered phylogenetically controlled comparisons. In 47 pairs, the aerobic prokaryotes have lower GC content than their anaerobic counterparts. Aerobic prokaryotes seem to have lower genomic GC content (Fig. 1b). However, two-tailed Wilcoxon signed ranks test showed that the difference was not statistically significant (*P* = 0.132). When the pairwise comparison is limited to the 80 pairs of bacteria, the difference between aerobes and anaerobes remains statistically insignificant (two-tailed Wilcoxon signed-rank test, *P* = 0.135). Our phylogenetically independent comparison of genomic GC content gave a result that is different from the nonphylogenetically controlled comparisons [10, 21–29], but consistent with two previous studies that have accounted for the phylogenetic relationship [24, 31].

Selective forces acting on non-synonymous sites might mask the specific effects of guanine oxidation within whole-genome sequences. For example, if codon GGG is mutated to TGG, this G to T mutation would be selected against because of the resulted change in the coded amino acid, from glycine to tryptophan. This exemplified mutation, even if occurs frequently, could not be fixed in evolution and so would not contribute to the evolution of GC content. In addition, the avoidance of oxidation-susceptible amino acids, of which the non-synonymous sites are AT-rich, might selectively increase the genomic GC content in aerobic prokaryotes [4]. The consequences of guanine oxidation, as a mutational bias, would be more accurately revealed by analysing the GC content of selectively neutral sequences or sequences under weak selection. Although the 4-fold degenerate sites (4FDS) might be under selection to maintain specific patterns of codon usage bias [52], they are by far the most common candidates for neutral or weakly selected sequences. Therefore, we performed pairwise comparison of the GC content at 4FDS. However, we did not find significant difference between aerobic prokaryotes and anaerobic prokaryotes (Fig. 1c, two-tailed Wilcoxon signed ranks test, *P* = 0.206).

Because horizontal gene transfer is extensive in prokaryotic evolution [60], the mutational force acting on the evolution of GC content in a lineage might be masked by the frequent horizontal transfer of DNA sequences with different GC content levels. The ideal genomic regions for comparison are sequences with orthologous relationships. For this reason, we compared the GC content of 4FDS within orthologous protein-coding genes. But still, we did not find significant difference between aerobic prokaryotes and anaerobic prokaryotes (Fig. 1d, two-tailed Wilcoxon signed ranks test, *P* = 0.334).

In addition to potential selective forces acting on non-synonymous sites and horizontal gene transfer, many other factors might increase the GC content of aerobes or decrease the GC content of anaerobes by specific mechanisms unrelated to changes in the oxygen requirement [8, 33]. GC-biased gene conversion has been widely observed as a driver of GC content increments [33, 34]. Organisms living at high temperatures tend to have higher GC contents in their structural RNA [35] and possibly in their whole-genome sequences (with debate, see [36–40]). G:C base pairs use more nitrogen and are energetically more costly than A:T base pairs; thus, AT-rich sequences may be favoured in non-nitrogen-fixing species and species living in challenging environments [8]. If guanine oxidation is a weak mutagenic force, then its effect on the evolution of GC content might be hidden by random combinations of these factors. Therefore, we propose that the relationship between oxygen requirement and GC content could be more accurately assessed if the oxygen requirement is the sole factor influencing the GC content that differs between each compared lineage. Although identifying all possible factors that influence the GC content of each species is impossible, distantly related species are more likely to differ in multiple factors that influence the GC content, whereas closely related aerobe-anaerobe pairs are more likely to differ only in the oxygen requirement, which is illustrated in Fig. 1a. In addition to the oxygen requirement, species 10 and species 11 are assumed to differ in the frequency of GC-biased gene conversion. The frequent GC-biased gene conversion in species 11 might lead to a much greater increase in the GC content relative to the decrease in GC content caused by guanine oxidation. If so, aerobic species 11 would have a higher GC content than anaerobic species 10. Thus, we examined whether the relationship between oxygen requirement and GC content depends on the divergence time between the paired lineages. The divergence time between a pair of lineages was represented by the identity of their 16S rRNA molecules. We found that, no matter which threshold was used to define the close relatedness, the differences in GC content between closely related aerobes and anaerobes were not statistically significant (two-tailed Wilcoxon signed ranks test, *P* > 0.05 for all the comparisons, Additional file 1: Table S1-S2).

### PGLS regression did not revealed difference between aerobes and anaerobes

The methods that integrate information on the phylogenetic relationships in testing evolutionary hypothesis are collectively termed as phylogenetic comparative methods. The most commonly used phylogenetic comparative method is PGLS. It is a special case of generalized least squares. With the assumption that shared ancestry would produce similar residuals from the least squares regression line, modified slope and intercept estimates are generated to account for interspecific autocorrelation due to common ancestors in cross-species data regression analysis [41]. By assigning organisms more preferring oxygen as 3 and that less, or not, preferring oxygen as 2, we performed PGLS regression analysis for the relationship between GC content and oxygen requirement, using oxygen requirement as the independent variable and GC content as the dependent variable. The slope of the regression would be positive if aerobiosis could increase GC content and it would be negative if aerobiosis could decrease GC content. By assigning the oxygen requirement of anaerobes and obligate anaerobes as 2 and that of aerobes and obligate aerobes as 3, we observed a positive slope, but the slope was not significant different from zero in either genomic GC content or GC content of 4FDS (*P* > 0.10 for both cases, Table 1). This result indicates no significant difference in GC content between the compared organisms, anaerobes and obligate anaerobes vs. aerobes and obligate aerobes. Next, we compared aerobes and anaerobes using PGLS regression analysis by assigning the oxygen requirement of anaerobes as 2 and that of aerobes as 3. Still, oxygen requirement was not significantly associated with either genomic GC content or GC content of 4FDS (*P* > 0.10 for both cases, Table 1). The PGLS regression analysis gave the same conclusion as the above pairwise comparisons.

**Table 1:** PGLS regression analysis of the relationship between aerobiosis and GC content

		whole-genome GC content			GC content at 4FDS			GC content at ZRS		
	No. of species	λ	Slope	*P*	λ	Slope	*P*	λ	Slope	*P*
Aerobes + obligate aerobes (3) vs. anaerobes + obligate anaerobes (2)	884	1.0	0.44	0.552	1.0	−0.25	0.882	1.0	0.42	0.153
Aerobe (3) vs. anaerobe (2)	799	1.0	0.34	0.661	1.0	−0.29	0.865	1.0	0.36	0.239
Obligate aerobes (3) vs. obligate anaerobes (2)	85	1.0	8.82	0.004	1.0	15.0	0.023	1.0	4.36	6 × 10^−4^
Obligate aerobes (3) vs. anaerobes (2)	333	1.0	5.21	0.001	1.0	7.87	0.037	1.0	4.50	0.005
Obligate aerobes (3) vs. aerobes (2)	546	1.0	1.47	0.014	1.0	3.19	0.014	1.0	2.13	0.324
Aerobes (3) vs. obligate anaerobes (2)	551	1.0	3.26	0.077	1.0	6.75	0.096	1.0	−0.68	0.294
Anaerobes (3) vs. obligate anaerobes (2)	338	1.0	0.86	0.407	1.0	3.50	0.143	1.0	−2.26	0.225

The numbers in parentheses are those assigned to each group of organisms in PGLS regression analyses. The data used in this analysis are deposited in Additional file 2. 4FDS: 4-fold degenerate sites, ZRS: zerofold redundant sites

### Obligate aerobes have higher GC contents

Finally, we put our hope in the extreme cases: obligate aerobes and obligate anaerobes. No matter mutational forces or selective forces associated with aerobiosis, they are expected to be stronger in obligate aerobes and obligate anaerobes than in aerobes and anaerobes. By assigning the oxygen requirement of obligate anaerobes as 2 and that of obligate aerobes as 3, we performed PGLS regression analysis and observed positive slopes in GC contents of both whole genome sequences and 4FDS (*P* < 0.05, Table 1). When assigning the oxygen requirement of obligate aerobes as the number 3 and that of anaerobes as 2, PGLS regression also showed significant difference in GC content between aerobes and obligate aerobes (*P* < 0.05, Table 1). Further PGLS regression analysis showed that obligate aerobes have significantly higher GC content than aerobes (*P* < 0.05, Table 1). A clear grade exists among the slopes of these three PGLS regression analyses: 8.82, 5.21, and 1.47 for whole-genome GC content and 15.0, 7.87, and 3.19 for 4FDS GC content (Table 1), indicating the existence of a grade in the GC content from obligate aerobes, aerobes, anaerobes and obligate anaerobes. In addition, we noticed that the slopes observed in 4FDS GC contents are steeper than those observed in whole-genome GC content (Table 1). No significant difference was observed when obligate anaerobes were compared with anaerobes or aerobes (*P* > 0.05, Table 1). Furthermore, we performed PGLS regression analysis on the GC content at zerofold redundant sites which were delineated by the second nucleotides of all codons except stop codons. At these sites, obligate aerobes have significantly higher GC content than obligate anaerobes and anaerobes (*P* < 0.01 for both cases, Table 1), but do not differ significantly from aerobes (*P* = 0.32). In addition, the slopes are flatter than those observed in whole-genome GC content and 4FDS (Table 1).

The pairwise comparison of closely related species is plain and easy to understand. However, compared with the advanced phylogenetic comparative methods, like PGLS, it does not make full use of the information in the phylogenetic tree. Thus, the pairwise comparison is recommended only when the sample size is more than sufficient. In this study, if obligate aerobes and aerobes were studied separately and obligate anaerobes and anaerobes were studied separately, we could get only seven pairs in which one partner is an obligate aerobe and only two pairs in which one partner is an obligate anaerobe. The sample size is apparently too small for a statistical analysis.

## Discussion

Guanine is more susceptible to oxidation than adenine, cytosine and thymine [2]. The replication of DNA containing damaged deoxyguanosines would cause G:C to T:A mutations [3]. The G:C to T:A transversions were widely observed as the dominant mutations in oxidatively damaged DNA, so they were considered the hallmark of oxidative damage to DNA [4–7]. Meanwhile, aerobic organisms are generally expected to experience more frequent oxidative damages than anaerobic organisms because of the unavoidable by-product, ROS, resulting from active consumption of oxygen. As a consequence, aerobiosis was generally believed to decrease GC content by oxidatively induced G:C to T:A transversions [8–10]. 16 years ago, Naya et al. [10] published a counterintuitive observation that aerobic prokaryotes had higher GC contents than anaerobic prokaryotes. This observation had been supported by nine later studies [21–29]. We noticed that all the comparisons did not account for the effects of common ancestors and so the results might be methodological artefacts resulting from the non-independence of the data [30]. For this reason, we performed pairwise comparison of the GC content between aerobic + obligate aerobic prokaryotes and anaerobic + obligate anaerobic prokaryotes that are adjacent in the phylogenetic tree (Fig. 1). As the difference between one aerobe-anaerobe pair is independent from the difference between any other pairs, the pairwise comparison is a phylogenetically controlled study. We did not observe significant difference in whole-genome GC content between aerobic prokaryotes and anaerobic prokaryotes (Fig. 1b, c, and d and Additional file 1: Figure S1). In addition, our PGLS regression analyses did not find significant differences between aerobic + obligate aerobic prokaryotes and anaerobic + obligate anaerobic prokaryotes, or between aerobic prokaryotes and anaerobic prokaryotes, either (Table 1). These results are consistent with two generally neglected studies that had accounted for the phylogenetic relationships [24, 31].

Obligate aerobes could not yield energy from fermentation. They generally live in high oxygen concentration and are subject to high levels of oxidative stress. We found that obligate aerobes have significantly higher GC content than aerobes, anaerobes, and obligate anaerobes (Table 1). The clear grade among the slopes indicates that the effect of oxidative stress is strong in obligate prokaryotes, moderate in aerobic prokaryotes, weak in anaerobic prokaryotes, and very weak in obligate anaerobic prokaryotes. Above all, no significant differences were observed between aerobes and anaerobes or between anaerobes and obligate anaerobes. Aerobiosis could increase GC content in evolution. However, the effect is not as strong as the nonphylogenetically controlled studies indicated, it could be observed only when the compared species differ in their oxygen requirement to a great extent.

To seek such a mutational force on GC content evolution, we attempted to control the potential selective force acting on the usage of amino acids by limiting the comparison of GC content within 4FDS. Overall, the results observed in the GC content of 4FDS were consistent with those obtained by calculating whole genome sequences, indicating that the evolution of GC content were mainly driven by a mutational force. The slopes obtained in 4FDS GC content are steeper than those obtained in whole-genome GC content, strengthening the idea that aerobiosis-associated GC content evolution is predominantly driven by a mutational force. Replication of DNA whose guanines have been oxidatively damaged would result in G:C to T:A mutations. Meanwhile, guanine oxidation can also occur before incorporation of the guanine nucleotide into DNA [14–16]. During replication, 8-oxodGTP would be incorporated at the position of thymidine, pairing with adenosine. In the next round of replication, the 8-oxoG would be paired with cytidine if it happens to switch into the *anti* conformation. The resulted change is a T to G mutation. This type of mutation has been clearly revealed by *E. coli* mutant strain lacking the MutT enzyme [15], which is responsible for repairing oxidatively damaged dGTP. The two mutational forces, after being decreased in some proportions by the repairing systems, might cancel each other out in their effects on the evolution of GC content to some extent. Our observation of higher GC content in obligate aerobes indicated that the incorporation of 8-oxodGTP should be a stronger mutational force than oxidative damage of guanine within DNA sequences in prokaryotes.

Compared with those of whole-genome sequences and 4FDS, the GC contents at zerofold redundant sites have weaker relationship with oxygen requirement. This is reasonable because all nucleotide substitutions at zerofold redundant sites would lead to amino-acid changes. The existence of the weak relationship indicates that amino acid usage is not only driven by the specific function of each protein, but also partially dictated by mutation pressure. This is consistent with a recent study that attempted to distinguish the evolutionary determinants of genome-wide nucleotide composition [42].

The antioxidant enzymes used by aerobes, like superoxide dismutase, have been identified in many obligate anaerobes [43–45]. Three enzymes, MutT, MutM and MutY, have well documented to be responsible for the repairing of oxidative damaged guanines [15]. Our preliminary survey showed that these enzymes are prevalent in anaerobic prokaryotes (Additional file 1: Table S3). Among the 85 anaerobic prokaryotes analysed in Fig. 1b, c, and d, genes encoding MutY, MutM and MutT have been detected in 67, 65, and 44 lineages, respectively. Meanwhile, in similar number of aerobic lineages (59, 56, and 46), the genes encoding these three enzymes have been detected. This result implicates the occasional occurrence of guanine oxidation in anaerobic prokaryotes. Apparently, obligate anaerobic prokaryotes and anaerobic prokaryotes suffered less frequent oxidative damages and thus experienced a weaker mutational force than aerobic prokaryotes and obligate aerobic prokaryotes.

The present study has also some implications on the sampling of evolutionary studies. In this study, the effect of aerobiosis was not observed when aerobic + obligate aerobic prokaryotes were compared with anaerobic + obligate anaerobic prokaryotes. However, if a study happens to have sampled mostly obligate prokaryotes, a significant difference would be observed. We suspect that the debate on the relationship between living temperature and GC content might come from different percentage of sampled organisms living in extreme temperatures [36–40]. Future studies paying particular attention on the organisms living in extreme temperatures might settle the controversy.

## Conclusions

By grouping aerobic and obligate aerobic prokaryotes together and anaerobic and obligate anaerobic prokaryotes together, our initial phylogenetically controlled analyses did not detect significant difference in GC content between aerobic prokaryotes and anaerobic prokaryotes. The result is different from nonphylogenetically controlled comparisons which always give a pattern of higher GC content in aerobes than anaerobes [10, 21–29], but consistent with two previous phylogenetically controlled studies [24, 31]. However, when obligate prokaryotes were studied separately, significant differences in GC content have been revealed. We suggest that the incorporation of 8-oxodGTP during DNA replication should be the main aerobiosis-associated mutational force, which is strong in obligate aerobes, moderate in aerobes, and weak in anaerobes and obligate anaerobes.

## Methods

In the Genomes Online Database (GOLD) [46], organisms are divided into ten categories according to their oxygen requirements: undefined, aerobe, anaerobe, facultative, facultative aerobe, facultative anaerobe, microaerophilic, microanaerobe, obligate aerobe, and obligate anaerobe. To avoid controversy, we retrieved only four categories: aerobe, anaerobe, obligate aerobe, and obligate anaerobe (access date: September 9, 2017). The GC contents of 2154 aerobic samples and 1758 anaerobic samples were obtained from the summary section of the homepage of each species or strain in the NCBI Genome database. In the nonphylogenetically controlled comparison, we used the average value to represent the GC content of species that had multiple strains consistent in oxygen requirements. In species of both aerobic and anaerobic strains, the GC content of each strain was considered an independent sample. The genome sequences of the paired species or strains were retrieved from the NCBI Genome database (ftp://ftp.ncbi.nlm.nih.gov/genomes/). The GC content used in the pairwise comparison was calculated from the downloaded genome sequences rather than retrieved directly from the NCBI Genome database. Although the GC content values from these two sources were not identical, they were highly similar. The regression equation was y = 0.9964x + 0.1388, and the R^2^ value was 0.9982.

In the phylogenetically controlled pairwise comparison, we want to compare each aerobic prokaryote with its closest anaerobic relative. Therefore, we selected all the species that included both aerobic and anaerobic strains. Then, from the remaining species, we selected all the genera that included both aerobic and anaerobic species. After that, we selected those families that included both aerobic and anaerobic genera. And so forth, we finally selected the classes that included both aerobic and anaerobic orders. Referring to the All-Species Living Tree [47], we roughly filtered out the species that were unlikely to be usable for pairwise comparison of closely related aerobes and anaerobes. For example, in Fig. 1, species 1, 2, 3 and 9 were discarded during the rough filtration of the samples. For the remaining samples, we constructed a neighbour-joining tree using the p-distance model integrated in the software MEGA7 with 16S rRNA [48]. The p-distance (pairwise nucleotide distance) is the proportion of sites at which nucleotide sequences differ divided by the total number of nucleotides compared. The bootstrap values were obtained with 1000 replications. For the poorly solved branches, we separately constructed their phylogenetic tree in the same way using 16S rRNA. In the four cases in which the phylogenetic relationships could not be resolved using 16S rRNA sequences, we constructed their phylogenetic trees using the *dnaj* gene sequence, which is another widely used phylogenetic marker [49–51]. Each difference in oxygen requirement between one pair of adjacent lineages was considered an event of evolutionary change in oxygen requirement (Fig. 1). The representative aerobic and anaerobic strains or species within each group were selected according to their branch lengths in the phylogenetic tree. For a comparative analysis of the GC content at 4FDS in orthologous genes, we retained only the genomes whose protein-coding sequences had been annotated. In total, our dataset included 85 aerobe-anaerobe pairs. For genomes in which the 16S rRNA gene annotations were not available, we identified the 16S rRNA genes by searching the genomes for the corresponding Rfam 13.0 profiles using Infernal (version 1.1.2) [52, 53]. We noticed that many bacterial genomes have not been fully assembled and some 16S rRNA sequences are fragmental. In the alignments of these 16S rRNA fragments, there are often large gaps not because of insertion/deletion occurred in evolution, but because of the incompleteness of the sequences. Both gaps and mismatches in the alignment are counted in the calculation of similarity, but only mismatches are counted in the calculation of identity. Identity is thus more solid than similarity in the comparison of fragmental 16S rRNA sequences. Therefore, we used the identity of 16S rRNA sequences to represent the divergence time between each pair of lineages. The sequences were aligned using ClustalW with its default parameters [54]. Orthologous genes between the paired lineages were first predicted by the reciprocal best blast hits and then screened using the program Ortholuge (version 0.8) using its default parameters [55, 56]. The thresholds of ratios 1 and 2 were both set to 0.8. Ortholuge is an ortholog-predicting method based on reciprocal best blast hits, and it improves the specificity of high-throughput orthologue predictions using an additional outgroup genome for reference. Ortholuge computes the phylogenetic distance ratios for each pair of orthologues that reflect the relative rate of divergence of the orthologues. Orthologues with a phylogenetic ratio that was significantly higher than that of the other orthologues in the genomes were considered incorrectly predicted and thus were discarded.

Published sequences of MutY, MutM, MutT from the bacterium *Escherichia coli* str. K-12 substr. MG1655 (NCBI taxonomy ID: 511145) and the archaea *Azotobacter vinelandii* DJ (NCBI taxonomy ID: 322710) were used in bi-directional BLASTP [57] (database: non-redundant protein sequences; default parameters) to search the candidate homologous proteins in the respective pairs of bacteria and archaea, respectively.

In phylogenetic regression analyses, the phylogenetic relationships among analysed species were obtained from the Genome Taxonomy Database [58]. The relationship between GC content and oxygen requirement was analysed with a phylogenetic regression approach using the ‘pgls’ function integrated in the package ‘ape’ (version 5.2) [59] in R (version 3.2.2) using Brownian model. Non-independence among continuous trait values due to their phylogenetic relatedness was measured by the phylogenetic signal, Pagel’s lambda (λ) value, which was also calculated using the ape package (version 5.2) [59]. In this study, all the λ values are close to 1, indicating that phylogenetically controlled analyses are required. To achieve a roughly symmetrical distribution of the analysed data, we used log-transformation. In the PGLS regression analyses, only fully sequenced genomes were used, including 506 aerobes, 293 anaerobes, 40 obligate aerobes, 45 obligate anaerobes. All these genomes were re-annotated by using the DFAST [60].

## Additional files

Additional file 1: **Figure S1.** and **Table S1.**-**S2.** Pairwise comparisons of GC content between aerobes + obligate aerobes and anaerobe + obligate anaerobes. **Table S3.** Presence and absence of genes coding enzymes responsible 8-oxoG repairing in the aerobic and anaerobic genome studied in Fig. 1b, c, and d. (DOCX 257 kb)

Additional file 2: The data generated and analysed during this study. (RAR 1282 kb)

## Abbreviations

4FDS: 4-fold degenerate sites
8-oxoG: 8-oxo-7,8-dihydro-guanosine
PGLS: Phylogenetic generalized least squares
ROS: Reactive oxygen species
