# Supplementary material for: Aerobic prokaryotes do not have higher GC contents than anaerobic prokaryotes, but obligate aerobic prokaryotes have
Source: BMC Evol Biol. 2019 Jan 28;19:35. doi: 10.1186/s12862-019-1365-8 (PMC6350292; doi:10.1186/s12862-019-1365-8)
Supplement: Supplementary file 1 — Figure S1. and Table S1.-S2. Pairwise comparisons of GC content between aerobes + obligate aerobes and anaerobe + obligate anaerobes. Table S3. Presence and absence of genes coding enzymes responsible 8-oxoG repairing in the aerobic and anaerobic genome studied in Fig. 1b, c, and d. (DOCX 257 kb) [file 12862_2019_1365_MOESM1_ESM.docx]

**Aerobiosis is not associated with GC content and G to T mutations are not the signature of oxidative stress in prokaryotes**

Sidra Aslam†, Xin-Ran Lan†, Bo-Wen Zhang, Zheng-Lin Chen and Deng-Ke Niu*

MOE Key Laboratory for Biodiversity Science and Ecological Engineering and Beijing Key Laboratory of Gene Resource and Molecular Development, College of Life Sciences, Beijing Normal University, Beijing 100875, China

†Contributed equally. *Author for Correspondence: Deng-Ke Niu, [dkniu@bnu.edu.cn](mailto:dkniu@bnu.edu.cn), [dengkeniu@hotmail.com](mailto:dengkeniu@hotmail.com)





**Fig. S1.** Pairwise comparison of GC content between aerobic and anaerobic prokaryotes. (A) Comparison of the GC content calculated from whole-genome sequences. (B) Comparison of GC content at the 4FDS of all protein-coding genes in each genome. (C) Comparison of GC content at the 4FDS of orthologous genes. The diagonal line represents cases in which aerobes and their paired anaerobes have the same GC content. Points above the line represent cases in which anaerobes have higher GC content than their paired aerobes, while points below the line indicate the reverse. All significance values were calculated using two-tailed Wilcoxon signed-rank tests.

**Table S1.** Relationship between GC content and aerobiosis is not dependent on divergence between compared lineages.

|  |  | *P* values of two-tailed Wilcoxon signed-rank tests | | |
| --- | --- | --- | --- | --- |
| 16S rRNA identity | Number of pairs | Whole genomes | 4FDS of all genes | 4FDS of orthologous genes |
| No limits | 85 | 0.132 | 0.206 | 0.334 |
| >0.8700 | 75 | 0.345 | 0.537 | 0.743 |
| >0.9033 | 65 | 0.673 | 0.771 | 0.971 |
| >0.9240 | 55 | 0.393 | 0.645 | 0.744 |
| >0.9414 | 45 | 0.358 | 0.546 | 0.697 |
| >0.9580 | 35 | 0.502 | 0.987 | 0.857 |
| >0.9770 | 25 | 0.427 | 0.968 | 0.696 |
| >0.9940 | 15 | 0.173 | 0.609 | 0.820 |

The results of this table were obtained using the same dataset as that used in figure 1B-1D. The divergence between each pair of aerobe-anaerobe lineages was represented by the identity of their 16S rRNA molecules. 4FDS: 4-fold degenerate sites.

**Table S2.** Relationship between GC content and oxygen requirement is not dependent on the divergence time between the compared aerobes and anaerobes.

|  |  | P values of two-tailed Wilcoxon signed-rank tests | | |
| --- | --- | --- | --- | --- |
| 16S rRNA identity | Number of pairs | Whole genomes | 4FDS of all genes | 4FDS of orthologous genes |
| No limits | 85 | 0.236 | 0.404 | 0.512 |
| >0.8600 | 75 | 0.252 | 0.438 | 0.568 |
| >0.9003 | 65 | 0.338 | 0.376 | 0.537 |
| >0.9150 | 55 | 0.436 | 0.633 | 0.700 |
| >0.9413 | 45 | 0.252 | 0.420 | 0.516 |
| >0.9580 | 35 | 0.342 | 0.831 | 0.935 |
| >0.9770 | 25 | 0.253 | 0.840 | 0.989 |
| >0.9940 | 15 | 0.140 | 0.865 | 1 |

The results of this table were obtained using the same dataset as that used in figure S1. The divergence time between each pair of lineages was represented by the identity of their 16S rRNA sequences. 4FDS: 4-fold degenerate sites.

**Table S3.** Presence and absence of genes coding enzymes responsible 8-oxoG repairing in the aerobic and anaerobic genome studied in figure 1B.

| Aerobes | MutY ID | MutM ID | MutT ID | Anaerobes | MutY ID | MutM ID | MutT ID |
| --- | --- | --- | --- | --- | --- | --- | --- |
| **Aeropyrum pernix K1** | **BAA79857.2** | **N/A** | **N/A** | **Ignicoccus islandicus DSM 13165** | **N/A** | **N/A** | **N/A** |
| **Haloarcula japonica DSM 6131** | **EMA30231.1** | **N/A** | **N/A** | **Halorhabdus tiamatea SARL4B** | **CCQ34673.1** | **N/A** | **N/A** |
| **Pyrolobus fumarii DSM 11204** | **N/A** | **N/A** | **N/A** | **Hyperthermus butylicus DSM 5456** | **N/A** | **N/A** | **N/A** |
| **Caldivirga maquilingensis IC-167** | **N/A** | **N/A** | **ABW00882.1** | **Pyrobaculum neutrophilum V24Sta** | **ACB39379.1** | **N/A** | **N/A** |
| **Metallosphaera sedula DSM 5348** | **N/A** | **N/A** | **N/A** | **Acidilobus saccharovorans 345-15** | **N/A** | **N/A** | **N/A** |
|  |  |  |  |  |  |  |  |
| Borrelia garinii PBr | N/A | N/A | N/A | Borrelia garinii BgVir | N/A | N/A | N/A |
| Brachyspira pilosicoli WesB | N/A | CCG56324.1 | N/A | Brachyspira pilosicoli 95/1000 | N/A | N/A | N/A |
| Candidatus Arthromitus sp. SFB-mouse-NL | N/A | N/A | AID45110.1 | Hathewaya proteolytica DSM 3090 | N/A | N/A | SHK20933.1 |
| Helicobacter hepaticus ATCC 51449 | AAP77839.1 | N/A | N/A | Sulfurimonas gotlandica GD1 | N/A | N/A | N/A |
| Enterococcus faecalis TX1322 | EEN75789.1 | EEN74060.1 | EEN75089.1 | Enterococcus faecalis PC1.1 | EFG20938.1 | EFG20082.1 | EFG19701.1 |
| Hydrogenobaculum sp. 3684 | N/A | N/A | N/A | Hydrogenivirga sp. 128-5-R1-1 | N/A | N/A | EDP76348.1 |
| Acinetobacter calcoaceticus RUH2202 | EEY77102.1 | EEY76114.1 | WP_003650682.1 | Acinetobacter calcoaceticus DSM 30006 | ENW02082.1 | ENV96957.1 | WP_005046891.1 |
| Enterococcus casseliflavus ATCC 12755 | EGC69149.1 | EGC69069.1 | N/A | Enterococcus cecorum DSM 20682 | EOX18359.1 | EOX18094.1 | EOX18022.1 |
| Acinetobacter sp. ATCC 27244 | WP_008942089.1 | EEH69940.1 | EEH67414.1 | Acinetobacter sp. SH024 | EFF85176.1 | N/A | EFF86391.1 |
| Legionella oakridgensis ATCC 33761 = DSM 21215 | AHE67433.1 | AHE66126.1 | AHE67417.1 | Legionella oakridgensis RV-2-2007 | WP_035894727.1 | ETO94186.1 | ETO92950.1 |
| Cyanothece sp. PCC 8801 | WP_012596301.1 | WP_012593515.1 | N/A | Cyanothece sp. PCC 7424 | ACK72892.1 | WP_015957033.1 | N/A |
| Acinetobacter tandoii DSM 14970 | EOR02666.1 | EOR11325.1 | EOR06560.1 | Acinetobacter parvus DSM 16617 | ENU35158.1 | ENU36525.1 | ENU36129.1 |
| Acinetobacter radioresistens WC-A-157 | EJO34063.1 | EJO35176.1 | EJO36016.1 | Acinetobacter radioresistens SH164 | EEY85960.1 | EEY86493.1 | EEY86922.1 |
| Pediococcus acidilactici DSM 20284 | N/A | EFL96085.1 | N/A | Pediococcus acidilactici MA18/5M | N/A | EHJ21700.1 | N/A |
| Streptococcus sp. I-P16 | AGY38117.1 | AGY38598.1 | WP_042507482.1 | Streptococcus sp. oral taxon 058 str. F0407 | EHI76570.1 | EHI76441.1 | EHI75812.1 |
| Simonsiella muelleri ATCC 29453 | EFG30186.1 | EFG30278.1 | WP_104930428.1 | Eikenella corrodens ATCC 23834 | WP_035579703.1 | EEG24808.1 | N/A |
| Thioalkalimicrobium cyclicum ALM1 | WP_013836113.1 | AEG31067.1 | AEG30983.1 | Hydrogenovibrio crunogenus XCL-2 | WP_011369542.1 | ABB42511.1 | ABB41188.1 |
| Tatumella morbirosei | WP_038019320.1 | KGD79672.1 | KGD70540.1 | Erwinia sp. Ejp617 | WP_014543294.1 | ADP10922.1 | WP_014542158.1 |
| Chlorobium sp. GBChlB | N/A | N/A | N/A | Chlorobium limicola DSM 245 | N/A | N/A | ACD89521.1 |
| Salmonella enterica Typhimurium U288 | AGK10744.1 | AGK11359.1 | AGK07755.1 | Salmonella enterica CVM N1543 | WP_001148960.1 | AJA99266.1 | AJB00792.1 |
| Geobacillus kaustophilus HTA426 | BAD74748.1 | BAD77013.1 | WP_081430700.1 | Geobacillus sp. T6 | KLR75247.1 | KLR74034.1 | : KLR75124.1 |
| Kosakonia sacchari SP1 | AHJ76626.1 | AHJ76056.1 | AHJ75241.1 | Shimwellia blattae DSM 4481 = NBRC 105725 | GAB82915.1 | AFJ48936.1 | AFJ48320.1 |
| Enterobacter cloacae subsp. cloacae ATCC 13047 | YP_003614769.1 | YP_003610643.1 | YP_003611409.1 | Enterobacter cloacae str. Hanford | EPR36676.1 | EPR40603.1 | EPR40969.1 |
| Cardiobacterium valvarum F0432 | EHM52616.1 | EHM55566.1 | EHM52678.1 | Dichelobacter nodosus VCS1703A | ABQ14153.1 | ABQ13185.1 | WP_012031285.1 |
| Thioalkalivibrio sp. K90mix | ADC72974.1 | ADC72491.1 | WP_041444279.1 | Halorhodospira halophila SL1 | WP_011813940.1 | ABM63074.1 | ABM62789.1 |
| Akkermansia sp. KLE1605 | KZA04470.1 | N/A | N/A | Akkermansia muciniphila ATCC BAA-835 | WP_081429226.1 | N/A | ACD03945.1 |
| Corynebacterium simulans | KXU17306.1 | AMO90045.1 | N/A | Corynebacterium striatum ATCC 6940 | WP_034657162.1 | EEI78654.1 | EEI79679.1 |
| Serratia nematodiphila DZ0503SBS1 | KFF88030.1 | KFF86612.1 | KFF87395.1 | Serratia marcescens WW4 | AGE19932.1 | AGE20558.1 | AGE16564.1 |
| Hyphomicrobium denitrificans 1NES1 | AGK59260.1 | AGK60032.1 | AGK59492.1 | Blastochloris viridis DSM 133 | WP_055038887.1 | ALK10939.1 | WP_055038597.1 |
| Herbaspirillum seropedicae SmR1 | ADJ65387.1 | ADJ65388.1 | ADJ61877.1 | Oxalobacter formigenes HOxBLS | WP_005878722.1 | N/A | EEO28283.1 |
| Corynebacterium glaucum | WP_095661179.1 | AQQ15565.1 | AQQ16285.1 | Corynebacterium riegelii | WP_083439864.1 | WP_101734449.1 | PLA12088.1 |
| Deinococcus deserti VCD115 | ACO45005.1 | ACO46592.1 | WP_083764275.1 | Deinococcus gobiensis I-0 | WP_043803938.1 | AFD24823.1 | AFD25033.1 |
| Microlunatus phosphovorus NM-1 | WP_049804651.1 | BAK34628.1 | BAK34938.1 | Pseudopropionibacterium propionicum F0230a | N/A | AFN47761.1 | N/A |
| Brevundimonas diminuta ATCC 11568 | EGF96263.1 | EGF95904.1 | EGF94404.1 | Brevundimonas diminuta 470-4 | EKY29904.1 | EKY25451.1 | EKY30467.1 |
| Brachybacterium faecium DSM 4810 | YP_003154739.1 | YP_003155244.1 | SLM96490.1 | Brachybacterium muris UCD-AY4 | EYT48306.1 | EYT47890.1 | EYT48134.1 |
| Streptomyces ghanaensis ATCC 14672 | WP_004984965.1 | WP_004982991.1 | WP_004983814.1 | Streptomyces griseoflavus Tu4000 | WP_040906486.1 | WP_004924876.1 | WP_040906203.1 |
| Mycoplasma genitalium M2321 | N/A | WP_010869400.1 | N/A | Mycoplasma mycoides subsp. capri LC str. 95010 | N/A | CBW54369.1 | N/A |
| Erysipelothrix rhusiopathiae ATCC 19414 | WP_003775993.1 | WP_003773930.1 | N/A | Holdemania filiformis DSM 12042 | WP_006059302.1 | EEF68019.1 | N/A |
| Weissella paramesenteroides ATCC 33313 | WP_002828104.1 | WP_002827125.1 | WP_002827293.1 | Weissella confusa LBAE C39-2 | WP_004560148.1 | WP_003609386.1 | N/A |
| Thermus sp. CCB US3 UF1 | N/A | N/A | N/A | Thermus scotoductus SA-01 | WP_015716129.1 | WP_015716350.1 | N/A |
| Pedobacter heparinus DSM 2366 | WP_012780308.1 | N/A | WP_015808230.1 | Solitalea canadensis DSM 3403 | WP_014681746.1 | N/A | N/A |
| Hylemonella gracilis ATCC 19624 | WP_006296347.1 | WP_006296346.1 | N/A | Rhodoferax ferrireducens T118 | WP_011463952.1 | WP_011463954.1 | N/A |
| Bifidobacterium animalis subsp. lactis ATCC 27673 | WP_022543034.1 | N/A | WP_022542670.1 | Bifidobacterium animalis BS 01 | WP_004269225.1 | N/A | WP_004218820.1 |
| Rubellimicrobium mesophilum DSM 19309 | WP_037280830.1 | WP_037278378.1 | WP_037281341.1 | Ketogulonicigenium vulgare Y25 | N/A | WP_013385127.1 | WP_013385432.1 |
| Gramella flava JLT2011 | WP_083644232.1 | N/A | N/A | Capnocytophaga granulosa ATCC 51502 | WP_016421104.1 | N/A | N/A |
| Hafnia alvei ATCC 51873 | EHM41494.1 | EHM40954.1 | EHM40556.1 | Edwardsiella ictaluri 93-146 | WP_015869723.1 | WP_015869540.1 | WP_015870160.1 |
| Endozoicomonas montiporae LMG 24815 | WP_034877657.1 | WP_034877867.1 | WP_034878457.1 | Hahella chejuensis KCTC 2396 | WP_011395280.1 | WP_011394548.1 | WP_011399580.1 |
| Shewanella algae BrY | WP_107110949.1 | WP_044735569.1 | WP_071237128.1 | Shewanella loihica PV-4 | WP_011864927.1 | WP_011867484.1 | WP_011867240.1 |
| Vibrio sinaloensis DSM 21326 | WP_008078686.1 | WP_008075749.1 | WP_008077360.1 | Photobacterium angustum S14 | WP_005371325.1 | WP_005371936.1 | WP_005371246.1 |
| Marinobacter algicola DG893 | WP_007153404.1 | WP_004094113.1 | N/A | Marinobacter hydrocarbonoclasticus ATCC 49840 | WP_014422533.1 | WP_011787153.1 | N/A |
| Lactobacillus lindneri DSM 20690 | N/A | KRN79102.1 | N/A | Lactobacillus ruminis ATCC 25644 | N/A | WP_003698067.1 | WP_003692397.1 |
| Thalassospira tepidiphila MCCC 1A03514 | WP_083997063.1 | WP_008888962.1 | WP_063088498.1 | Pararhodospirillum photometricum DSM 122 | WP_014415400.1 | WP_041794715.1 | WP_014413678.1 |
| Sulfurimonas autotrophica DSM 16294 | WP_041675244.1 | N/A | N/A | Sulfurimonas denitrificans DSM 1251 | N/A | N/A | N/A |
| Myroides xuanwuensis | WP_083543826.1 | N/A | N/A | Myroides odoratus DSM 2801 | WP_002989805.1 | WP_013729756.1 | N/A |
| Jannaschia sp. CCS1 | WP_011453521.1 | WP_011457323.1 | WP_044006205.1 | Rhodobacter sp. SW2 | WP_040671357.1 | WP_008027546.1 | WP_008030881.1 |
| Acinetobacter baumannii MDR-TJ | AFI93720.1 | N/A | N/A | Acinetobacter baumannii TCDC-AB0715 | ADX94253.1 | ADX93719.1 | N/A |
| Streptococcus mitis B6 | N/A | N/A | N/A | Streptococcus mitis bv. 2 str. F0392 | WP_000886162.1 | WP_001114612.1 | N/A |
| Streptococcus gallolyticus subsp. gallolyticus ATCC BAA-2069 | N/A | N/A | N/A | Streptococcus gallolyticus UCN34 | WP_012962467.1 | WP_012962296.1 | N/A |
| Bifidobacterium longum E18 | N/A | N/A | N/A | Bifidobacterium longum DJO10A | WP_007053737.1 | N/A | N/A |
| Bacillus anthracis str. Ames | N/A | N/A | N/A | Bacillus sp. X1 | WP_038539936.1 | WP_038536654.1 | N/A |
| Klebsiella michiganensis M1 | WP_032751251.1 | PLL97330.1 | WP_014227925.1 | Klebsiella pneumoniae PittNDM01 | WP_002916628.1 | WP_004173907.1 | WP_004145942.1 |
| Desulfovibrio termitidis HI1 | N/A | N/A | N/A | Desulfovibrio vulgaris str. 'Miyazaki F' | WP_012613039.1 | WP_015946081.1 | WP_015946013.1 |
| Thermus aquaticus Y51MC23 | N/A | N/A | N/A | Thermus oshimai JL-2 | AFV76934.1 | WP_016330204.1 | N/A |
| Pasteurella multocida gallicida X73 | WP_032854096.1 | WP_005757285.1 | WP_005717611.1 | Mannheimia succiniciproducens MBEL55E | WP_011199499.1 | WP_041640022.1 | N/A |
| Nitrosococcus oceani ATCC 19707 | N/A | WP_002813868.1 | WP_002813165.1 | Marichromatium purpuratum 984 | WP_005221932.1 | WP_005222228.1 | N/A |
| Thermaerobacter marianensis DSM 12885 | N/A | N/A | N/A | Sulfobacillus acidophilus DSM 10332 | AEW05064.1 | AEW06053.1 | N/A |
| Trichodesmium erythraeum IMS101 | N/A | N/A | N/A | Microcoleus sp. PCC 7113 | AFZ21845.1 | WP_015183729.1 | N/A |
| Oceanobacillus iheyensis HTE831 | WP_011065302.1 | WP_011066556.1 | N/A | Amphibacillus xylanus NBRC 15112 | WP_015009366.1 | WP_015009677.1 | N/A |
| Microcystis aeruginosa NIES-843 | N/A | WP_012265637.1 | WP_012263846.1 | Halothece sp. PCC 7418 | WP_015226308.1 | WP_015224517.1 | N/A |
| Myxococcus xanthus DK 1622 | WP_011552066.1 | WP_011555883.1 | WP_011553782.1 | Anaeromyxobacter dehalogenans 2CP-1 | WP_012633246.1 | WP_012631715.1 | WP_012633461.1 |
| Idiomarina loihiensis L2TR | WP_011235211.1 | WP_011233507.1 | WP_011233707.1 | Psychromonas ingrahamii 37 | WP_011768796.1 | WP_011768486.1 | WP_011769556.1 |
| Herpetosiphon aurantiacus DSM 785 | N/A | N/A | N/A | Oscillochloris trichoides DG-6 | WP_006563537.1 | WP_006563731.1 | N/A |
| Coraliomargarita akajimensis DSM 45221 | WP_013044428.1 | WP_013043291.1 | N/A | Opitutus terrae PB90-1 | WP_083767695.1 | WP_012375191.1 | N/A |
| Candidatus Phytoplasma mali | N/A | WP_012504227.1 | N/A | Spiroplasma mirum ATCC 29335 | N/A | WP_025317903.1 | N/A |
| Aeromonas veronii B565 | WP_005354841.1 | WP_005356002.1 | WP_085941982.1 | Frischella perrara PEB0191 | WP_039105400.1 | WP_039103514.1 | WP_039103381.1 |
| Bordetella parapertussis Bpp5 | WP_010927127.1 | WP_003808494.1 | WP_003815027.1 | Bordetella petrii DSM 12804 | N/A | N/A | N/A |
| Paenibacillus larvae DSM 25430 | AHD04582.1 | WP_024095116.1 | N/A | Paenibacillus senegalensis JC66 | WP_010272725.1 | WP_010272019.1 | N/A |
| Phaeobacter inhibens 2.10 | N/A | N/A | N/A | Phaeobacter inhibens DSM 16374 | WP_027248420.1 | WP_027248257.1 | WP_014876346.1 |
| Borrelia duttonii Ly | N/A | N/A | N/A | Spirochaeta africana DSM 8902 | N/A | WP_014455159.1 | N/A |
| Beutenbergia cavernae DSM 12333 | WP_015883762.1 | WP_015882106.1 | WP_015884236.1 | Arcanobacterium haemolyticum DSM 20595 | WP_013169525.1 | WP_013170287.1 | N/A |

Incomplete genome sequencing and low similarity between some analysed genomes with the reference genomes, bacterium *Escherichia coli* str. K-12 substr. MG1655 and the archaea *Azotobacter vinelandii* DJ, might cause some existing genes undetectable. However, these potential errors are expected to affect the results in aerobes and anaerobes to the same extent. Five pairs of archaea were marked in bold letters.
